# Comparing the Effectiveness of Endoscopic Surgeries With Intensity-Modulated Radiotherapy for Recurrent rT3 and rT4 Nasopharyngeal Carcinoma: A Meta-Analysis

**DOI:** 10.3389/fonc.2021.703954

**Published:** 2021-07-26

**Authors:** Zhouying Peng, Yumin Wang, Yaxuan Wang, Ruohao Fan, Kelei Gao, Hua Zhang, Weihong Jiang

**Affiliations:** Department of Otolaryngology Head and Neck Surgery, Xiangya Hospital, Central South University, Changsha, China

**Keywords:** recurrent nasopharyngeal carcinoma, endoscopic surgery, intensity-modulated radiotherapy, survival outcome, meta-analysis

## Abstract

**Background:**

This meta-analysis aimed to compare the efficacy of intensity-modulated radiotherapy (IMRT) and endoscopic surgery (ES) for high T-stage recurrent nasopharyngeal carcinoma (NPC).

**Methods:**

Relevant studies were retrieved in six databases from 02/28,2011 to 02/28,2021. The 2-year, 3-year, 5-year overall survival (OS) rates and 2-year disease-free survival (DFS) rates were calculated to compare the survival outcomes of the two treatments of IMRT and ES. Combined odds ratios (ORs) and 95% confidence interval (C Is) were measured as effect size on the association between high T-stage and 5-year OS rates.

**Results:**

A total of 23 publications involving 2,578 patients with recurrent NPC were included in this study. Of these, 1611 patients with recurrent rT3-4 NPC were treated with ES and IMRT in 358 and 1,253 patients, respectively. The combined 2-year OS and 5-year OS rates for the two treatments were summarized separately, and the 2-year OS and 5-year OS rate for ES were 64% and 52%, respectively. The 2-year OS and 5-year OS rate for IMRT were 65% and 31%, respectively. The combined 2-year DFS rates of IMRT and ES were 60% and 50%, respectively. Combined ORs and 95% confidence intervals for 5-year survival suggest that ES may improve survival in recurrent NPC with rT3-4. In terms of complications, ES in the treatment of high T-stage recurrent NPC is potentially associated with fewer complications.

**Conclusions:**

The results of our study suggest that ES for rT3-4 may be a better treatment than IMRT, but the conclusion still needs to be sought by designing more studies.

## Introduction

Nasopharyngeal carcinoma (NPC) is a type of squamous head and neck cancer with variable geographic distribution, with the highest incidence in Southeast Asia. Its main treatment modality is radiotherapy (1–4). With the development of diagnostic and treatment techniques, the 5-year OS rate of NPC reaches 50% to 64%, but 10% to 20% of patients still experience recurrence after the first treatment and improvement of their disease (5). According to National Comprehensive Cancer Network (NCCN) guidelines, surgical excision of the lesion or local radiotherapy is recommended for resectable head and neck squamous carcinoma after recurrence that has been treated with radiotherapy. Chemotherapy alone is usually reserved for palliative patients who are not candidates for radiotherapy or surgery (5, 6).

The 5-year survival rate for salvage nasopharyngectomy for resectable recurrent NPC is 40% to 60%, compared with 8% to 36% for patients with local recurrence treated with recourse radiotherapy and often with severe complications, such as multiple cranial nerve palsies, osteonecrosis, and internal carotid artery dissection (7). For patients with locally advanced rT3-4, endoscopic surgical resection of the lesion requires a high level of surgical skill on the lead surgeons, and the probability of subsequent complications is higher than that of early-stage patients if they are treated with re-radiotherapy (7, 8). The survival and prognosis studies of ES and IMRT for recurrent NPC have been reported in the literatures (5, 9, 10), but there is no literature comparing the efficacy of the two treatment modalities for locally advanced recurrent rT3-4 NPC.

For recurrent rT3-4 NPC, whether ES should be used or IMRT should be performed is unclear. There needs to be an evidence-based summary of which treatment is better for these patients to provide some basis for clinicians’ treatment decisions. Therefore, we conducted a meta-analysis to synthesize the best currently available data to compare the efficacy and safety of ES and IMRT for the treatment of recurrent rT3-4 NPC.

## Materials And Methods

### Search Strategy

We conducted a literature search on several medical databases, including Pubmed, Embase, Web of Science, Cochrane, and two Chinese databases (CNKI and Wanfang). The studies published from February 1, 2011, to February 1, 2021. The search strategy was predefined according to the following Medical Subject Headings (MeSH) and free terms: “recurrent” or “recurrence”, “nasopharyngeal carcinoma”, “endoscopy surgery”, or “intensity modulated radiotherapy”. Publications in a language other than Chinese and English were excluded. At the same time, the references of included papers were examined for potentially eligible studies.

### Inclusion and Exclusion Criteria

The eligible studies in this meta-analysis meet the following criteria: (a) histologically confirmed residual and/or recurrent NPC patients with T-stage information; (b) initial treatment is at least one cycle of radiotherapy, with or without concurrent chemotherapy; (c) patients with recurrent NPC treated with endoscopic surgery or IMRT with or without chemotherapy; (d) the outcome of publication studies include randomized controlled studies with 2-year survival or 3-year survival or 5-year survival, 2-year DFS or 3-year LCR, retrospective studies, case series reports, and so on; (e) the number of patients with stage rT3 and rT4 is 5 or more than; (f) when multiple studies report the same sample, the one with the most complete data from the available studies will be selected.

The following criteria were used to exclude studies: (a) case reports, reviews, and meta-analyses; (b) studies without rT3 and rT4 stage cases; (c) various studies with incomplete survival rates.

### Data Extraction and Assessment

**Figure 1:**
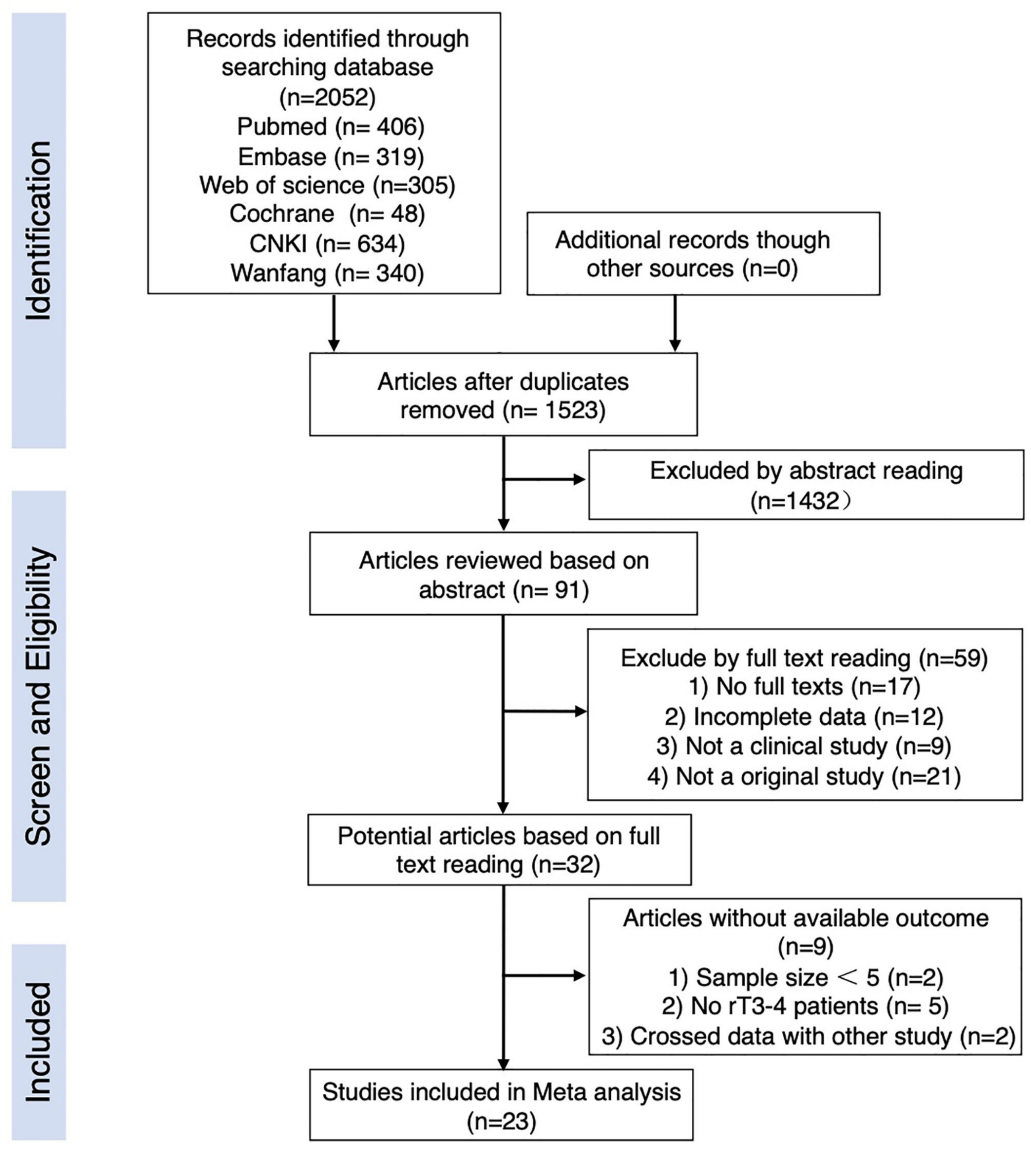
Flowchart of the process of trial selection.

All retrieved publications were read by two researchers simultaneously and independently. In case of disagreement between the two researchers, a detailed discussion by a third researcher was required. After the relevant publications were identified, each publication was screened based on its title and abstract, and inappropriate publications were eliminated. The retained publications were then reviewed in their entirety to determine their final inclusion in this study. The literature review workflow is shown in **Figure 1**. Finally, valid data were extracted from the publications that met the criteria for inclusion in this study. The extracted information included: sample characteristics, tumor T-stage, specific information on treatment, post-treatment follow-up time, and survival rate. If no survival rate information was required in the publications, they were reanalyzed as accurately as possible based on the data in the articles. **Tables 1** and **2** list the details of the studies included in this study.

**Table 1:** Main characteristics of included studies on endoscopic surgery.

Authors	Year	Publication language	No. of patients	M/F	rT classifications		Margins	2-year OS (%)		5-year OS (%)		2-year DFS (%)		MINORS
					rT1-2	rT3-4	+/-	Overall	rT3-4	overall	rT3-4	overall	rT3-4	
Castelnuovo et al. (11)	2013	English	27	—	13	14	3/27	—	78.6	72.5	—	—	57.1	9
You et al. (9)	2015	English	72	54/18	59	13	—	93.3	84.6	77.1	76.9	92.8	47.2	12
Wong et al. (7)	2017	English	15	9/6	0	15	6/9	66.7	66.7	—	50.0	40.0	40.0	9
Weng et al. (12)	2017	English	36	26/10	17	19	—	68.3	52.6	—	—	63.6	48.0	10
Liu et al. (13)	2017	English	91	71/20	43	48	—	64.8	53.7	38.3	—	57.5	57.5	11
Tang et al. (14)	2019	English	55	44/11	45	10	4/51	—	90.0	—	—	—	30.0	8
Zou et al. (10)	2015	English	92	70/22	79	13	—	91.3	—	78.1	48.5	—	—	10
Wong et al. (8)	2019	English	12	—	0	12	—	—	—	50.0	—	—	—	9
Li et al. (15)	2020	English	189	132/57	97	92	32/157	82.2	—	43.6	47.2	—	—	9
Sun et al. (16)	2015	Chinese	71	53/18	37	34	17/20	74.0	41.1	39.0	—	60.5	—	10
Chen and Qiu (17)	2015	Chinese	96	72/24	38	58	52/44	68.0	51.7	—	—	—	—	9
Liu et al. (5)	2021	English	96	—	66	30	6/90	89.9	—	73.8	—	81.8	—	21

CI, confidence interval; M, male; F, female; OS, overall survival; DFS, disease-free survival.

**Table 2:** Main characteristics of included studies on IMRT.

Authors	Year	Publication language	No. of patients	M/F	rT classifications		ReRT mean GTV dose (Gy)	rT3-4 NPC’ OS (%)			2-year DFS (%)	3-year LCR (%)	MINORS
					rT1-2	rT3-4		2-year	3-year	5-year			
Qiu et al. (18)	2012	English	70	56/14	30	40	70.0 (50–77.4)	64.7	—	—	62.0	—	8
Han et al. (19)	2012	English	239	182/57	59	180	69.9 (61.7–78.7)	—	—	35.1	—	—	10
Hua et al. (20)	2012	English	151	122/29	29	122	70.4 (62.1–77.6)	—	42.6	34.4	—	88.5	9
Chen et al. (21)	2013	English	54	44/10	11	43	70.0(49.8–76.6)	65.9	—	—	—	—	9
Tian et al. (22)	2013	English	251	195/56	53	198	70.7(61.1–79.7)	—	—	32.4	—	—	11
Karam et al. (23)	2016	English	27	20/7	21	6	54.0 (39.0–97.0)	—	23.0	—	—	8.0	10
Chan et al. (24)	2016	English	38	31/7	0	38	—	—	47.2	—	—	44.3	8
Ng et al. (25)	2017	English	33	—	0	33	—	—	63.8	—	—	49.2	8
Tian et al. (26)	2017	English	245	49/196	0	245	70.0 (60.1–78.7)	—	—	27.5	—	—	10
Kong et al. (27)	2018	English	184	133/51	64	120	66.7 (42.0–77.0)	—	42.9	—	—	—	9
Zhang et al. (28)	2018	Chinese	44	33/11	21	23	66.0 (54.0–70.0)	—	33.8	—	—	—	8
You et al. (9)	2015	English	72	18/54	59	13	—	—	46.1	46.1	53.8	—	12
Zou et al. (10)	2015	English	218	173/45	57	161	—	—	—	28.8	—	—	10
Liu et al. (5)	2021	English	100	72/28	69	31	—	—	—	—	—	—	21

CI, confidence interval; M, male; F, female; OS, overall survival; DFS, disease-free survival; LCR, local control rate.

All the included articles were assessed according to the Methodological Index for Non-Randomized Studies (MINORS). The MINORS instrument has eight dimensions assessing study objective, patients enrollment, data collection, endpoints definition, endpoints assessment, follow-up period, lost to follow-up, and sample size calculation. The high MINORS scores indicate good quality. The maximum ideal score is 16 for nonrandomized studies and 24 for controlled studies. Specific MINORS scores are detailed in **Tables 1** and **2**.

### Statistical Analysis

We analyzed the obtained data for survival rate, OR, and other factors by using Review Manager 5.3. For survival rate, we merged the rate values and performed heterogeneity tests. I (2) > 50% was defined as significant heterogeneity. A random effect model was adopted and sub-analyses were made when heterogeneity existed among study results. Otherwise, a fixed-effect model was adopted to merge survival rate values and 95% CIs. Calculation results were presented as forest plots. OR is the ratio of the survival rates in the case of surgery group to the prognosis in the case of radiotherapy group. OR > 1 indicates that the patients with surgery have a better prognosis than the patients with radiotherapy. For the studies from which we could obtain survival data, we made a funnel plot to describe publication bias using S.E. of rate as the abscissa and mean rates as the ordinate.

Symmetry of the funnel plot was tested by linear regression models (Begg’s method and Egger’s method) in STATA 12.0 to evaluate publication bias. For the studies from which we could obtain survival of endoscopic surgery group and IMRT group, OR values were combined and heterogeneity tests was analyzed using Review Manager 5.3. The relationship between treatment methods and patients survival rate was shown by pooled OR. We calculated the OR values and performed to analyze P <0.05 was considered statistically significant. We use Engauge Digitizer 12.1 to calculate from Kaplan-Meier (K-M) Curve of published article (29).

## Results

### Search Results and Study Characteristics

A total of 2,052 publications were captured though the initial systematic research of literature published between February 28, 2011, and February 28,2021, among which 1,078 were found in English databases, and 974 were found in Chinese databases. While reviewing the titles and abstracts, 91 of them were selected for full-text reading and 68 of them were excluded for the reasons shown in **Figure 1**. Twenty-three papers were finally left for our analysis.

The 23 publications meeting the inclusion criteria include a total of 2,578 patients diagnosed with recurrent NPC. Twelve articles investigated endoscopic surgery for recurrent NPC covered a total of 852 patients, including 494 patients with stage recurrent rT1-2 NPC and 358 patients with stage rT3-4. Fourteen studies investigated IMRT for recurrent NPC covered a total of 1,726 patients, including 473 patients with stage recurrent rT1-2 NPC and 1,253 patients with stage rT3-4, respectively. Three of the 23 studies were comparative studies of the efficacy of endoscopic surgery versus IMRT for recurrent NPC. The range of sample size was 12 to 251 with a mean of 112, sample size for endoscopic treatment, and IMRT ranging from 12 to 189 with a mean of 71 and 27 to 251 with a mean of 123, respectively. We used MINORS to assess the quality of the studies, and in all publications included in this paper, the average MINORS score was 10 (range,8-21) (**Tables 1** and **2**), so the quality of these studies is acceptable.

### Comparison of OS in Patients Between Endoscopic Surgery and IMRT

**Figure 2:**
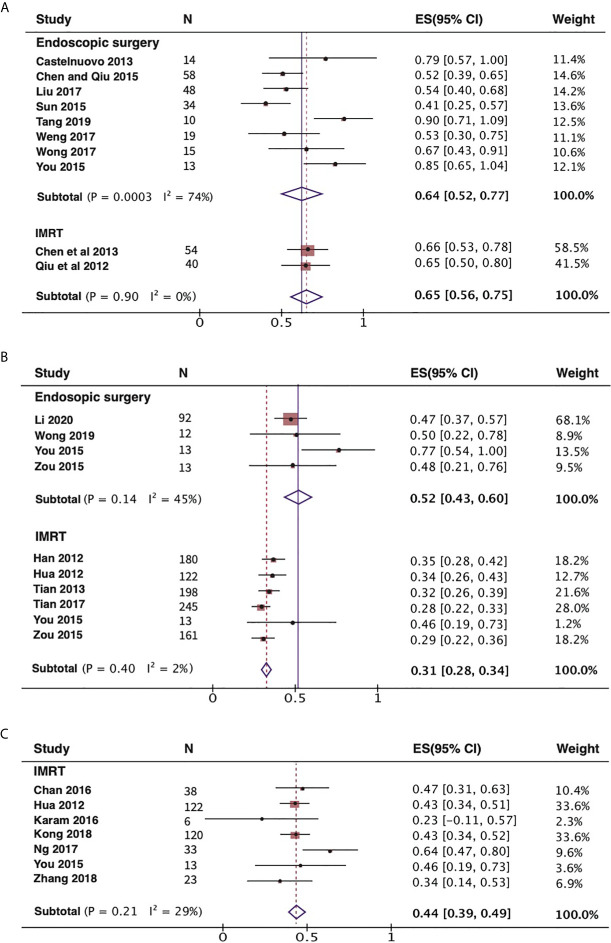
Forest plot displaying the meta-analysis of OS in the endoscopic surgery and IMRT group with recurrent rT3-4 NPC. **(A)** Meta-analysis of 2-year OS rates. **(B)** Meta-analysis of 5-year OS rates. **(C)** Meta-analysis of 3-year OS rates for IMRT group. NPC, nasopharyngeal carcinoma; IMRT, intensity-modulated radiotherapy; OS, overall survival.

First step meta-analysis, we performed on OS rate. We compared the 2-year and 5-year OS rates of endoscopic surgery and IMRT with or without chemotherapy for recurrent rT3-4 NPC, respectively (**Figures 2A, B**). For these patients, the 2-year OS rate was 64% (95% CI, 52%–77%, I^2^ = 74%, P= 0.0003), the 5-year OS rate was 52% (95% CI, 43%–60%, I^2 ^= 45%, P= 0.14). Although the patients undertaking IMRT had better 2-year OS rate than those with endoscopic surgeries. However, there is just a little difference between them. In terms of 5-year OS rate, the patients undertaking ES had better survival experience than those with IMRT. In studies of IMRT for recurrent NPC, most investigators use the 3-year OS rate to express the effectiveness of the treatment method, so we also analyzed the 3-year OS rate of the IMRT group as shown in **Figure 2C**. Then, we found that ES achieved better 5-year OS rate than IMRT’s 3-year OS rate for recurrent rT3-4 NPC (52% *vs* 44%).

### Association Between Treatment and OS, DFS, and LCR

**Figure 3:**
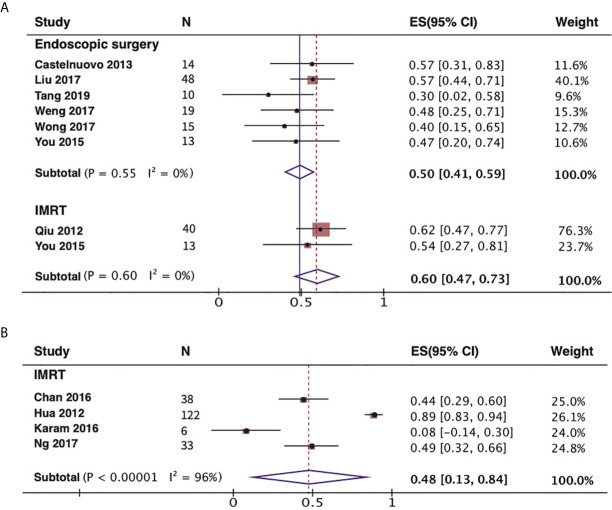
Forest plot of DFS and LCR in the endoscopic surgery and/or IMRT group with recurrent rT3-4 NPC. **(A)** Meta-analysis of 2-year DFS rates. **(B)** Meta-analysis of 3-year LCR for IMRT group. NPC, nasopharyngeal carcinoma; IMRT, intensity-modulated radiotherapy; DFS, disease-free survival; LCR, local control rate.

**Figure 4:**
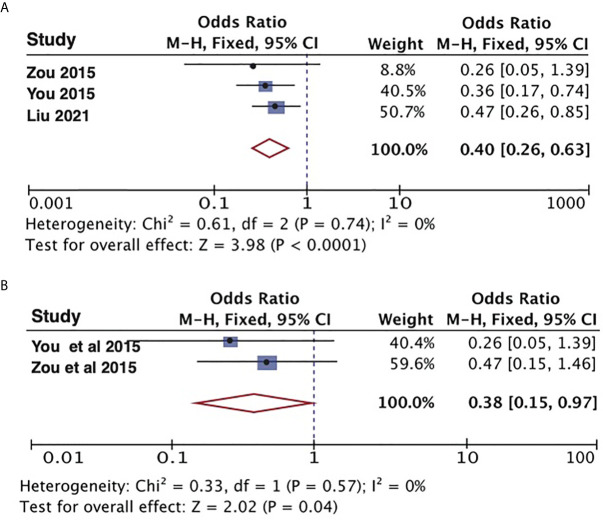
Meta-analysis the impact on 5-year OS rate with endoscopic surgery and IMRT. **(A)** Results of the overall cases. **(B)** Results of the recurrent rT3-4 NPC. NPC, nasopharyngeal carcinoma; OS, overall survival.

The 2-year disease-free survival (DFS) rate are comparable between ES and IMRT for recurrent rT3-4 NPC. Although the 2-year DFS rate was higher in patients with recurrent NPC treated with IMRT than in those treated with ES, 60%(95% CI,47%–73%, I^2^ = 0%, P= 0.6) and 50% (95% CI,41%–59%, I^2^ = 0%, P= 0.55), respectively, the confidence interval was wider than for ES (**Figure 3A**). We also combined the 3-year local control rate (LCR) of recurrent rT3-4 NPC treated with IMRT as shown in **Figure 3B**, 48%(95% CI,13%-84%, I^2^ = 96%, P<0.00001). However, this metric does not have enough data for comparison in ES. As shown in **Figure 4**, endoscopic surgery for recurrent NPC is more advantageous compared with IMRT, also in recurrent cases with high T-stage, but the confidence interval was wider, probably related to the relatively small number of included literatures.

I (2) > 50% indicates a high heterogeneity in the analytical results, so we further investigated possible causes of bias and heterogeneity. Meta-regression analysis showed that size of studies (≥15 cases or not) was a correlative factor of heterogeneity. Subgroup meta-analysis was then performed (**Supplementary Figure 1**). If we exclude the studies which size of study<15 cases, then combined the 2-year OS rate, N=5, the results obtained are shown in **Supplementary Figure 1A**. Similar results were also observed in meta-regression analyses for 3-year LCR for IMRT group. Combining the 3-year LCR rate of two studies with similar case numbers, we can obtain results as shown in **Supplementary Figure 1B**.

### Comparison of Complications

**Figure 5:**
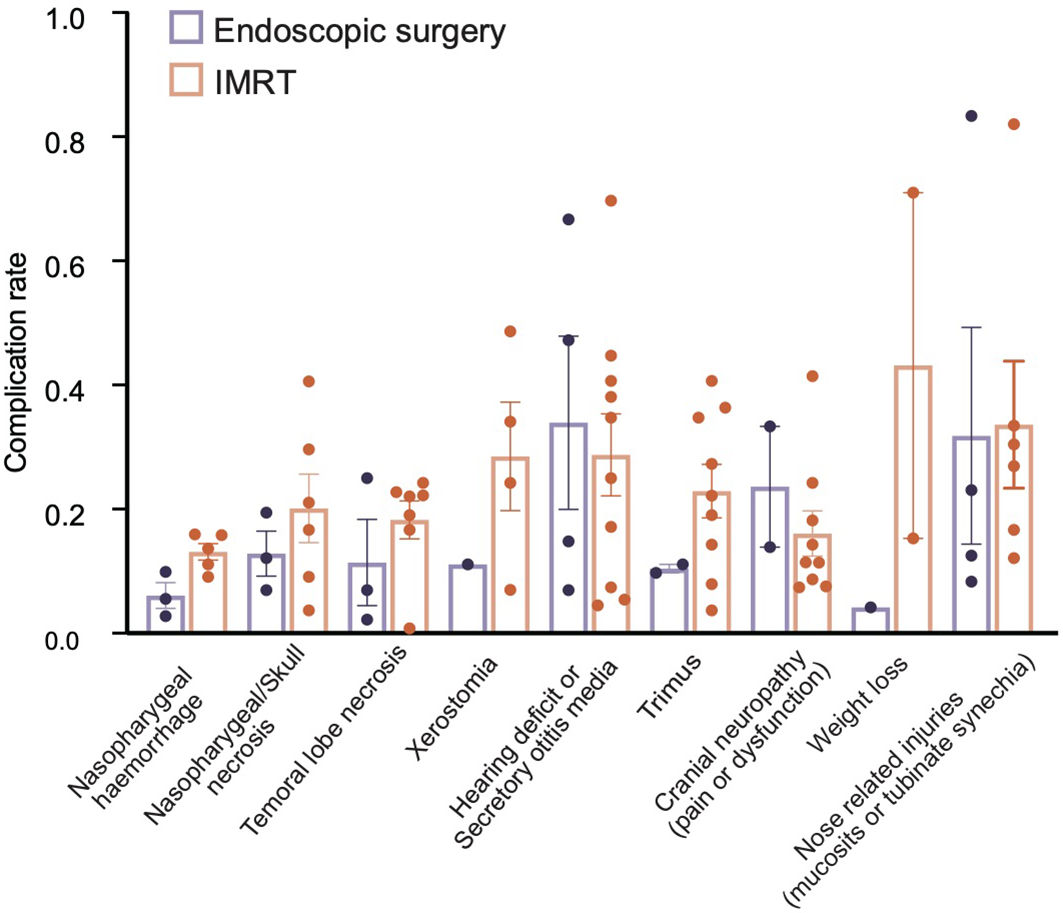
Comparison of the combined incidence of major complications of endoscopic surgery and IMRT.

We combined the occurrence of complications of the two treatments in 23 papers respectively; as shown in **Figure 5**, the incidence of most complications after IMRT for patients with recurrent NPC is higher than that of ES. Also, among the 13 publications on IMRT, three reported 23 cases of dysphagia in a total of 125 patients and 68 patients with radiation encephalopathy in another publications of 239 patients. Four publications reported tissue damage to the face and neck in 20 of a total of 288 patients treated with re-radiotherapy. Postoperative hemorrhage and wound infection have become complications specific to ES for recurrent NPC.

### Publication Bias

**Figure 6:**
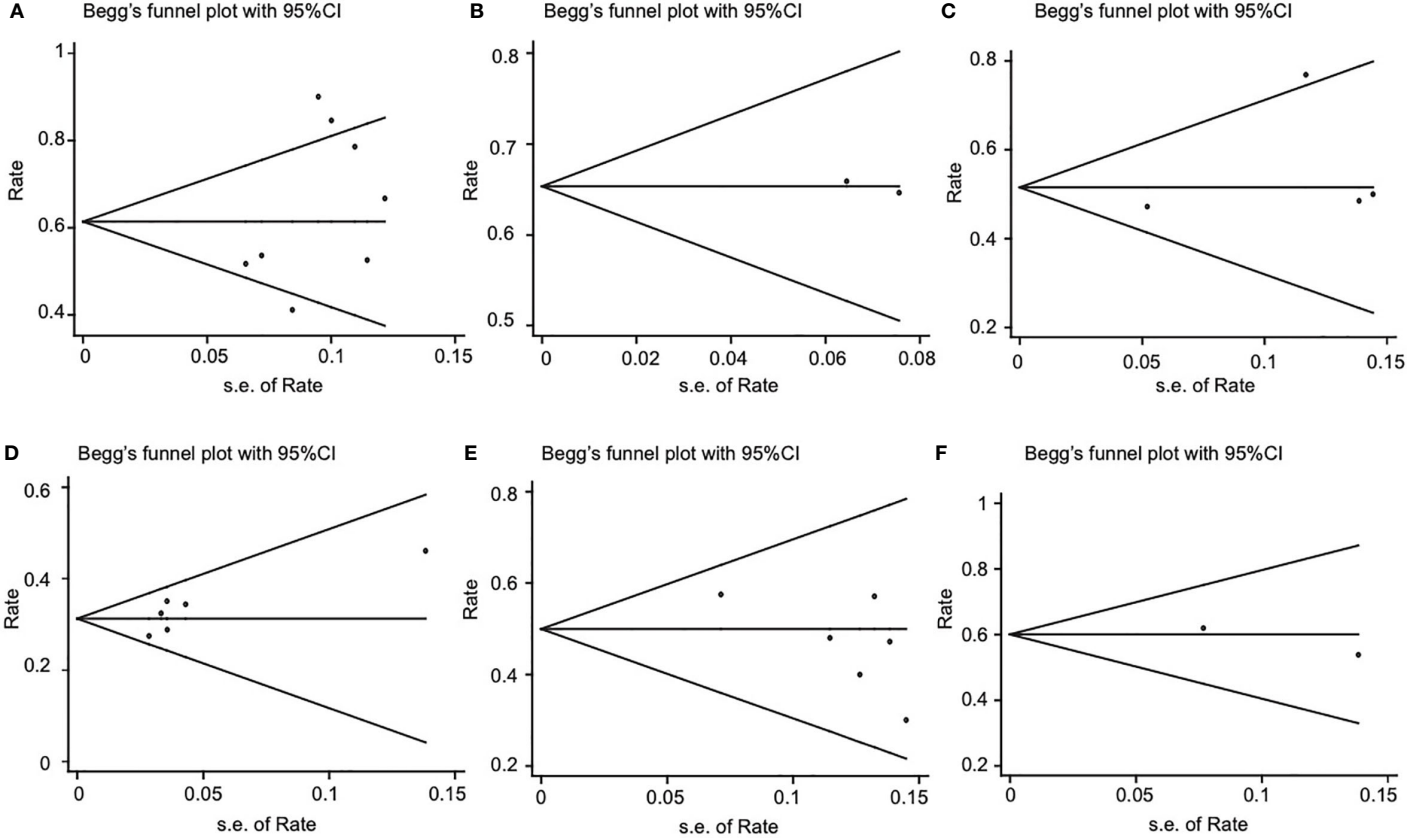
Funnel plots for publication bias of 2-year OS, 5-year OS, 2-year DFS. **(A)** 2-year OS of endoscopic surgery. **(B)** 2-year OS of IMRT. **(C)** 5-year OS of endoscopic surgery. **(D)** 5-year OS of IMRT. **(E)** 2-year DFS of endoscopic surgery. **(F)** 2- year DFS of IMRT. OS, overall survival; DFS, disease-free survival.

Publication bias was evaluated using Begg’s test. As shown in **Figure 6**, the funnel plot did not indicate any evidence of publication bias for endoscopic surgery’s 2-year OS (p = 0.458) and 5-year OS (p = 0.497); IMRT 2-year OS (p = 0.317) and 5-year OS (p = 0.188); endoscopic surgery’s 2- year DFS (p = 0.091).

## Discussion

Nasopharyngeal carcinoma is sensitive to radiotherapy but more prone to recurrence. Tumor size, pathological staging, tumor necrosis, tumor stage, the presence of lymph node metastasis, or distant metastasis have important effects on patient survival and recurrence, and the prognosis of high T-stage NPC is relatively poor. There is no accepted unified standard treatment for the recurrent NPC (7, 8, 26). IMRT with or without chemotherapy is a more widely used treatment modality at present. With the development of endoscopic surgical techniques and the deepening of nasocranial base surgeons’ understanding of the anatomy of the nasopharynx in recent years, endoscopic surgery for recurrent NPC has been well developed and has good efficacy (5, 9, 10). Our study synthesized the publications in the last 10 years for patients with high T-stage recurrent NPC and compared the survival rates of ES and IMRT as well as some other indicators to determine the prognosis. This article is the first meta-analysis comparing the efficacy of ES with IMRT for recurrent rT3-4 NPC only, and it is also the article with the largest number of cases of recurrent rT3-4 NPC combined. In conclusion, endoscopic surgery for recurrent rT3-4 NPC was superior to IMRT, in terms of both 2-year and 5-year OS rates, and had a lower complication rate.

Radiotherapy is the traditional treatment modality for NPC. For the first occurrence of NPC, especially if the tumor is relatively limited in growth and has not yet involved the skull base structures and important blood vessels or nerves, radiotherapy with or without synchronous or neoadjuvant chemotherapy can achieve good results (30). In recurrent NPC, reirradiation with conventional external beam techniques have yielded largely unsatisfactory results with high rates of late complications even with transition from 2-D to 3-D conformal techniques. IMRT allows for conformation of multiple small beamlets to irregularly shaped tumors, such as NPC (31). Compared with conventional radiotherapy techniques, IMRT is better at reducing complications, but the toxic effects of radiotherapy are still present, and the efficacy is not conclusive. A meta-analysis by Leong et al, which investigated 12 studies including 1768 patients, concluded that the 5-year local failure-free survival of IMRT for recurrent NPC was 72%, 5-year distant failure-free survival was 85%, and 5-year OS was 41% (31). From the above survival data, the long-term survival rate of recurrent NPC treated with IMRT still needs to be improved. With the development of endoscopic surgical techniques and anatomical studies, endoscopic surgery for recurrent NPC is increasingly performed. In 2005, Yoshizaki et al. introduced ES to treat patients with recurrent NPC by selecting four different approaches according to the T-stage of the tumor and the site of growth (32). Yang et al. summarized 23 papers; they combined survival outcomes for recurrent NPC treated with ES and reported that 1-year, 2-year, and 5-year OS rates were 97%, 92%, and 73%, respectively (33). These data are more favorable compared with the same type of data for IMRT.

In the treatment of recurrent NPC, surgery not only leads to similar survival and prognosis as IMRT but also has better outcomes in terms of complications and severity compared with those who undergo IMRT. A review of previous publications shows that the T-stage of NPC has a significant impact on survival outcomes, with patients with T1-2 tumors, who had almost all better OS, DFS, and LCR than patients with T3-4. Combining the 2-year OS rate according to rT1 to rT4 stage were 100%, 87%, 78%, and 38%, respectively, indicating a trend toward diminishing OS rate correlated with staging of tumors (33). You et al. reported improved survival in ES cases compared to IMRT in rT1 to rT3 patients in a subgroup analysis (9). For patients with recurrent rT3-4 NPC, surgery provides better targeted protection of important nerves and blood vessels than conventional IMRT. Of course, the abovementioned results presuppose that the surgeon should be very familiar with the structures of the nasopharynx and related skull base anatomical regions and proficient in endoscopic surgical techniques, at the same time, because many patients will have different degrees of radiation injury after their first radiation treatment, such as secretory otitis media, head and facial pain, difficulty in opening the mouth, slurred speech, and so on, and even more serious post-radiation complications, such as carotid artery hemorrhage and cranial nerve injury can occur in some patients. Patients in this category cannot receive re-radiation if they relapse, regardless of their T-stage. Although chemotherapy has a certain effect on controlling metastatic tumors, it cannot completely replace radiotherapy and can only be used to assist in killing the residual tumor cells. Therefore, for such patients, endoscopic surgery may be offered as a new hope.

In recent years, some surgeons believe that open surgery provides better clarity for late recurrent NPC, and a better safety margin can be obtained with an open view. In contrast, endoscopic surgery seems to be more suitable for the treatment of early recurrent NPC. In fact, a meta-analysis study of endoscopic and open surgery for recurrent NPC by investigators showed that for patients with rT3 stage, the 2-year OS rate was 67% for endoscopic surgery compared with 53% for open surgery. For patients with rT4, there was no difference in the 2-year OS rate between the two treatments, which were both 35% (34). Although there are no comparative results for 5-year OS rates, it is at least clear that modern high-definition endoscopic surgery is not inferior in the management of tumor safety margins. Also compared with the occurrence of postoperative complications, such as infection and bleeding, endoscopic surgery is more advantageous than open surgery.

There are some limitations worth noting of this study that should be acknowledged. First, all but one of the publications included in this study were retrospective studies and lacked randomized controlled trials, increasing the risk of bias. Second, although three papers on endoscopic treatment and IMRT for recurrent NPC at the same institution were included in this study, the literature was mostly from different medical centers. IMRT was acceptable, although the prognosis and survival of ES were actually related to the surgical technique and even surgical equipment of the physicians in the medical institutions and the presence of these objective factors may affect the results of this meta-analysis. Furthermore, some results in this study were analyzed with less literature included, which can introduce bias to the analysis results. Finally, because some of the original clinical data were not available, some of the data obtained by statistical methods may not be accurate enough, which ultimately affects the results of the analysis.

## Conclusions

Our study showed that, compared with IMRT, endoscopic surgery was a more effective treatment modality in managing patients with recurrent rT3-4 NPC. However, there is still insufficient evidence to suggest that ES can replace IMRT, but only to provide some support for the choice of perhaps more appropriate treatment. Ultimately, RCT will need to be designed to corroborate the current view.

## Data Availability Statement

The raw data supporting the conclusions of this article will be made available by the authors, without undue reservation.

## Author Contributions

WJ, HZ, ZP, and YMW conceived and designed the study. ZP, YXW, RF, and KG performed the analysis, prepared the figures and tables, and wrote the main manuscript. All authors contributed to the article and approved the submitted version.

## Funding

This Research was funded by the National Natural Science Foundation of China (81770985); the Hunan Province Graduate Education Innovation Project (CX20200386); and the Independent Exploration and Innovation Project for Graduate Students of Central South University (506021703). The funders had no role in study design, data collection and analysis, decision to publish, or preparation of the manuscript.

## Conflict of Interest

The authors declare that the research was conducted in the absence of any commercial or financial relationships that could be construed as a potential conflict of interest.

## Publisher’s Note

All claims expressed in this article are solely those of the authors and do not necessarily represent those of their affiliated organizations, or those of the publisher, the editors and the reviewers. Any product that may be evaluated in this article, or claim that may be made by its manufacturer, is not guaranteed or endorsed by the publisher.

## References

[1] Chen YP Chan ATC Le QT Blanchard P Sun Y Ma J. Nasopharyngeal Carcinoma. Lancet (2019) 394(10192):64–80. 10.1016/S0140-6736(19)30956-0 31178151

[2] Xu T Tang J Gu M Liu L Wei W Yang H. Recurrent Nasopharyngeal Carcinoma: A Clinical Dilemma and Challenge. Curr Oncol (2013) 20(5):e406–19. 10.3747/co.20.1456 PMC3805410 24155638

[3] Wang Y Mo Y Gong Z Yang X Yang M Zhang S. Circular RNAs in Human Cancer. Mol Cancer (2017) 16(1):25. 10.1186/s12943-017-0598-7 28143578 PMC5282898

[4] Peng Z Wang Y Wang Y Jiang S Fan R Zhang H. Application of Radiomics and Machine Learning in Head and Neck Cancers. Int J Biol Sci (2021) 17(2):475–86. 10.7150/ijbs.55716 PMC7893590 33613106

[5] Liu YP Wen YH Tang J Wei Y You R Zhu XL. Endoscopic Surgery Compared With Intensity-Modulated Radiotherapy in Resectable Locally Recurrent Nasopharyngeal Carcinoma: A Multicentre, Open-Label, Randomised, Controlled, Phase 3 Trial. Lancet Oncol (2021) 22(3):381–90. 10.1016/S1470-2045(20)30673-2 33600761

[6] Hao CY Hao SP. The Management of Rnpc: Salvage Surgery Vs. Re-irradiation. Curr Oncol Rep (2020) 22(9):86. 10.1007/s11912-020-00949-0 32642860

[7] Wong EHC Liew YT Abu Bakar MZ Lim EYL Prepageran N. A Preliminary Report on the Role of Endoscopic Endonasal Nasopharyngectomy in Recurrent rT3 and rT4 Nasopharyngeal Carcinoma. Eur Arch Otorhinolaryngol (2017) 274(1):275–81. 10.1007/s00405-016-4248-2 27520568

[8] Wong EHC Liew YT Loong SP Prepageran N. Five-Year Survival Data on the Role of Endoscopic Endonasal Nasopharyngectomy in Advanced Recurrent rT3 and Rt4 Nasopharyngeal Carcinoma. Ann Otol Rhinol Laryngol (2020) 129(3):287–93. 10.1177/0003489419887410 31701754

[9] You R Zou X Hua YJ Han F Li L Zhao C. Salvage Endoscopic Nasopharyngectomy Is Superior to Intensity-Modulated Radiation Therapy for Local Recurrence of Selected T1-T3 Nasopharyngeal Carcinoma – A Case-Matched Comparison. Radiother Oncol (2015) 115(3):399–406. 10.1016/j.radonc.2015.04.024 25987536

[10] Zou X Han F Ma WJ Deng MQ Jiang R Guo L. Salvage Endoscopic Nasopharyngectomy and Intensity-Modulated Radiotherapy Versus Conventional Radiotherapy in Treating Locally Recurrent Nasopharyngeal Carcinoma. Head Neck (2015) 37(8):1108–15. 10.1002/hed.23719 24764204

[11] Castelnuovo P Nicolai P Turri-Zanoni M Battaglia P Bolzoni Villaret A Gallo S. Endoscopic Endonasal Nasopharyngectomy in Selected Cancers. Otolaryngol Head Neck Surg (2013) 149(3):424–30. 10.1177/0194599813493073 23764963

[12] Weng J Wei J Si J Qin Y Li M Liu F. Clinical Outcomes of Residual Or Recurrent Nasopharyngeal Carcinoma Treated With Endoscopic Nasopharyngectomy Plus Chemoradiotherapy or With Chemoradiotherapy Alone: A Retrospective Study. PeerJ (2017) 5:e3912. 10.7717/peerj.3912 29038762 PMC5637710

[13] Liu J Yu H Sun X Wang D Gu Y Liu Q. Salvage Endoscopic Nasopharyngectomy for Local Recurrent or Residual Nasopharyngeal Carcinoma: A 10-Year Experience. Int J Clin Oncol (2017) 22(5):834–42. 10.1007/s10147-017-1143-9 28601934

[14] Tang IP Ngui LX Ramachandran K Lim LY Voon PJ Yu KL. A 4-Year Review of Surgical and Oncological Outcomes of Endoscopic Endonasal Transpterygoid Nasopharyngectomy in Salvaging Locally Recurrent Nasopharyngeal Carcinoma. Eur Arch Otorhinolaryngol (2019) 276(9):2475–82. 10.1007/s00405-019-05522-5 31227870

[15] Li W Lu H Wang H Zhang H Sun X Hu L. Salvage Endoscopic Nasopharyngectomy in Recurrent Nasopharyngeal Carcinoma: Prognostic Factorsand Treatment Outcomes. Am J Rhinol Allergy (2021) 35(4):458–66. 10.1177/1945892420964054 33019819

[16] Sun X Liu J Wang H Yu H Wang J Li H. Endoscopic Nasopharyngectomy for Recurrent Nasopharyngeal Carcinoma: A Review of 71 Patients and Analysis of Theprognostic Factors. Zhonghua Er Bi Yan Hou Tou Jing Wai Ke Za Zhi (2015). 50(11):890–95. 26887992

[17] Chen Z Qiu Q. Analysis of Clinical Eifcacy and the Quality of Life After Endoscopic Nasopharyngectomy for Residual or Recurrent Nasopharyngela Carcinoma. Zhonghua Er Bi Yan Hou Tou Jing Wai Ke Za Zhi (2015) 50(11):896–903. 26887993

[18] Qiu S Lin S Tham IW Pan J Lu J Lu JJ. Intensity-Modulated Radiation Therapy in the Salvage of Locally Recurrent Nasopharyngeal Carcinoma. Int J Radiat Oncol Biol Phys (2012) 83(2):676–83. 10.1016/j.ijrobp.2011.07.006 22024207

[19] Han F Zhao C Huang SM Lu LX Huang Y Deng XW. Long-Term Outcomes and Prognostic Factors of Re-Irradiation for Locally Recurrent Nasopharyngeal Carcinoma Using Intensity-Modulated Radiotherapy. Clin Oncol (R Coll Radiol) (2012) 24(8):569–76. 10.1016/j.clon.2011.11.010 22209574

[20] Hua YJ Han F Lu LX Mai HQ Guo X Hong MH. Long-Term Treatment Outcome of Recurrent Nasopharyngeal Carcinoma Treated With Salvage Intensity Modulated Radiotherapy. Eur J Cancer. (2012) 48(18):3422–8. 10.1016/j.ejca.2012.06.016 22835782

[21] Chen HY Ma XM Ye M Hou YL Xie HY Bai YR. Effectiveness and Toxicities of Intensity-Modulated Radiotherapy for Patients With Locally Recurrent Nasopharyngeal Carcinoma. PLoS One (2013) 8(9):e73918. 10.1371/journal.pone.0073918 24040115 PMC3769398

[22] Tian YM Tian YH Zeng L Liu S Guan Y Lu TX. Prognostic Model for Survival of Local Recurrent Nasopharyngeal Carcinoma With Intensity-Modulated Radiotherapy. Br J Cancer (2014) 110(2):297–303. 10.1038/bjc.2013.715 24335924 PMC3899759

[23] Karam I Huang SH McNiven A Su J Xu W Waldron J. Outcomes After Reirradiation for Recurrent Nasopharyngeal Carcinoma: North American Experience. Head Neck (2016) 38(Suppl 1):E1102–9. 10.1002/hed.24166 26451876

[24] Chan OS Sze HC Lee MC Chan LL Chang AT Lee SW. Reirradiation With Intensity-Modulated Radiotherapy for Locally Recurrent T3 to T4 Nasopharyngeal Carcinoma. Head Neck (2017) 39(3):533–40. 10.1002/hed.24645 27898191

[25] Ng WT Ngan RKC Kwong DLW Tung SY Yuen KT Kam MKM. Prospective, Multicenter, Phase 2 Trial of Induction Chemotherapy Followed by Bio-Chemoradiotherapy for Locally Advanced Recurrent Nasopharyngeal Carcinoma. Int J Radiat Oncol Biol Phys (2018) 100(3):630–8. 10.1016/j.ijrobp.2017.11.038 29413277

[26] Tian YM Huang WZ Yuan X Bai L Zhao C Han F. The Challenge in Treating Locally Recurrent T3-4 Nasopharyngeal Carcinoma: The Survival Benefit and Severe Late Toxicities of Re-Irradiation With Intensity-Modulated Radiotherapy. Oncotarget (2017) 8(26):43450–7. 10.18632/oncotarget.15896 PMC5522160 28427216

[27] Kong F Zhou J Du C He X Kong L Hu C. Long-Term Survival and Late Complications of Intensity-Modulated Radiotherapy for Recurrent Nasopharyngeal Carcinoma. BMC Cancer (2018) 18(1):1139. 10.1186/s12885-018-5055-5 30453915 PMC6245884

[28] Zhang HH Zhang XW Jiang H. Clinical Efficacy and Prognostic Factors of Locally Recurrent Nasopharyngeal Carcinoma With Intensity- Modulated Radiotherapy. J Shanghai Jiao Tong Uni (2018) 38(6):662–9.

[29] Shi X Chen Q Wang F. Mesenchymal Stem Cells for the Treatment of Ulcerative Colitis: A Systematic Review and Meta-Analysis of Experimental and Clinical Studies. Stem Cell Res Ther (2019) 10(1):266. 10.1186/s13287-019-1336-4 31443677 PMC6708175

[30] Kong L Lu JJ. Reirradiation of Locally Recurrent Nasopharyngeal Cancer: History, Advances, and Promises for the Future. Chin Clin Oncol (2016) 5(2):26. 10.21037/cco.2016.03.19 27121886

[31] Leong YH Soon YY Lee KM Wong LC Tham IWK Ho FCH. Long-Term Outcomes After Reirradiation in Nasopharyngeal Carcinoma With Intensity-Modulated Radiotherapy: A Meta-Analysis. Head Neck (2018) 40(3):622–31. 10.1002/hed.24993 29130584

[32] Yoshizaki T Wakisaka N Murono S Shimizu Y Furukawa M. Endoscopic Nasopharyngectomy for Patients With Recurrent Nasopharyngeal Carcinoma at the Primary Site. Laryngoscope (2005) 115(8):1517–9. 10.1097/01.MLG.0000165383.35100.17 16094136

[33] Yang J Song X Sun X Liu Q Hu L Yu H. Outcomes of Recurrent Nasopharyngeal Carcinoma Patients Treated With Endoscopic Nasopharyngectomy: A Meta-Analysis. Int Forum Allergy Rhinol (2020) 10(8):1001–11. 10.1002/alr.22552 32452124

[34] Li G Wang J Tang H Han R Zhao Y Wang X. Comparing Endoscopic Surgeries With Open Surgeries in Terms of Effectiveness and Safety in Salvaging Residual or Recurrent Nasopharyngeal Cancer: Systematic Review and Meta-Analysis. Head Neck (2020) 42(11):3415–26. 10.1002/hed.26397 33463833

