# Supplementary figures and images for: Comparing the Effectiveness of Endoscopic Surgeries With Intensity-Modulated Radiotherapy for Recurrent rT3 and rT4 Nasopharyngeal Carcinoma: A Meta-Analysis
Source: Front Oncol. 2021 Jul 26;11:703954. doi: 10.3389/fonc.2021.703954 (PMC8350726; doi:10.3389/fonc.2021.703954)

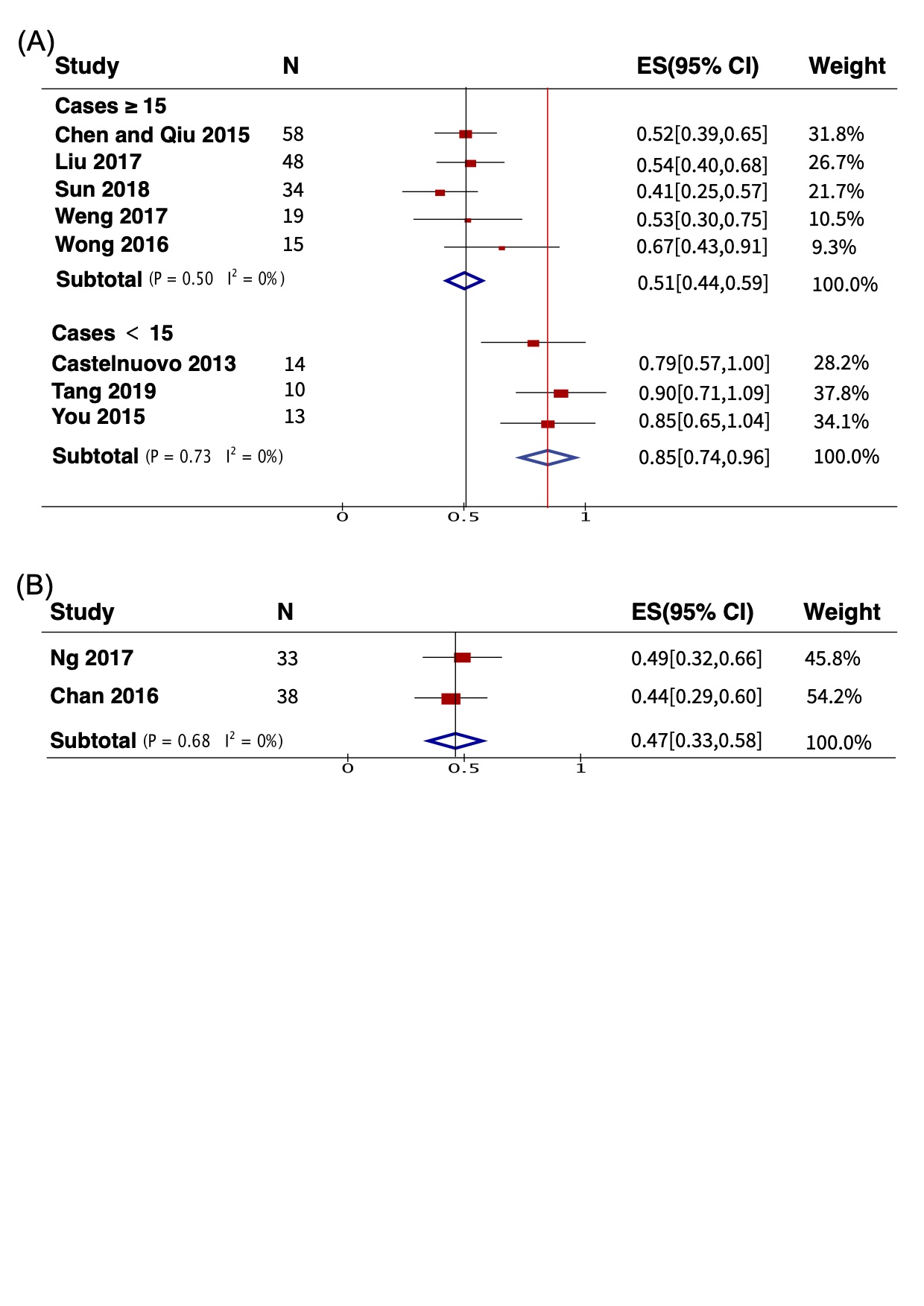

Supplement: Supplementary file 1 [file Image_1.jpeg]
